# Framework for network modularization and Bayesian network analysis to investigate the perturbed metabolic network

**DOI:** 10.1186/1752-0509-5-S2-S14

**Published:** 2011-12-14

**Authors:** Hyun Uk Kim, Tae Yong Kim, Sang Yup Lee

**Affiliations:** 1 Metabolic and Biomolecular Engineering National Research Laboratory, Department of Chemical and Biomolecular Engineering (BK21 program), Center for Systems and Synthetic Biotechnology, Institute for the BioCentury, Korea Advanced Institute of Science and Technology (KAIST), Daejeon 305-701, Republic of Korea; 2 BioInformatics Research Center, KAIST, Daejeon 305-701, Republic of Korea; 3 Department of Bio and Brain Engineering and BioProcess Engineering Research Center, KAIST, Daejeon 305-701, Republic of Korea

## Abstract

**Background:**

Genome-scale metabolic network models have contributed to elucidating biological phenomena, and predicting gene targets to engineer for biotechnological applications. With their increasing importance, their precise network characterization has also been crucial for better understanding of the cellular physiology.

**Results:**

We herein introduce a *f*ramework for network *m*odularization and *B*ayesian network analysis (FMB) to investigate organism’s metabolism under perturbation. FMB reveals direction of influences among metabolic modules, in which reactions with similar or positively correlated flux variation patterns are clustered, in response to specific perturbation using metabolic flux data. With metabolic flux data calculated by constraints-based flux analysis under both control and perturbation conditions, FMB, in essence, reveals the effects of specific perturbations on the biological system through network modularization and Bayesian network analysis at metabolic modular level. As a demonstration, this framework was applied to the genetically perturbed *Escherichia coli* metabolism, which is a *lpdA* gene knockout mutant, using its genome-scale metabolic network model.

**Conclusions:**

After all, it provides alternative scenarios of metabolic flux distributions in response to the perturbation, which are complementary to the data obtained from conventionally available genome-wide high-throughput techniques or metabolic flux analysis.

## Background

Genome-scale metabolic network models have proved to be useful in aiding biological and biotechnological research by providing large-scale predications, and their scope of applications is still expanding [1]. They predict gene targets to manipulate in metabolic engineering for overproduction of industrially valuable chemicals [2,3], or predict drug targets for drug discovery [4-7]. These metabolic network models are a coherently organized set of metabolites whose relationships are stoichiometrically defined and mass-balanced through biochemical reactions [8]. They are often simulated with constraints-based flux analysis that employs optimization-based techniques with an objective function, typically maximizing biomass formation rate, and constraints that reflect biophysical conditions affecting the cellular physiology [9,10]. Due to their importance, the number of currently reported genome-scale metabolic networks is constantly increasing, spanning archaea, bacteria, and eukaryotes [1].

Reconstruction of metabolic network models has been accompanied by systematic characterization of their network properties for appropriate analysis of the prediction outcomes [11]. Some noteworthy constraints-based approaches for elucidating correlations among intracellular reactions include flux coupling analysis [12], assessment of alternative sets of reactions for the optimal solutions [13], uniform random sampling [14], and elucidation of metabolic core reactions [15]. As an extension of these studies, correlations among metabolic modules that behave in a synchronized manner in the solution space allowed under the specific condition attracted attention for further investigations. For this, application of clustering (or modularization) and Bayesian network (BN) analysis to metabolic network models and their flux data was considered in this study in order to reveal other complementary aspects of the cell that are statistically and biologically important under the perturbation condition – modular-level behaviour of metabolism.

Clustering has succeeded in revealing key components and their correlations, contributing to our understanding of biological systems through a systemic concept of module [16,17]. Modularization of biological network, which is based on clustering algorithms, elucidates topological design of biological system, from which evolutionary and functional clues can be inferred [18,19]. Metabolic network can also be clustered based on the characteristics of its metabolic fluxes, which are defined as the cellular phenotype derived from interplays of many factors, including transcription, translation, enzyme activity, and metabolite concentration, and revealed as reaction rates at steady state in metabolic pathways [20-22]. Therefore, modularization of metabolic network based on pattern of metabolic flux variation would cluster biochemical reactions, which can then be considered as functional units, and simplify subsequent computational analyses.

BN analysis is increasingly adopted to extract useful information from messy high-throughput biological data [23,24]. It is a probabilistic graphical model that reveals changes that each variable causes in another variables, so called *causal relationship*, in the form of a directed acyclic graph, and has successfully been applied to reverse engineering of various biological networks from large datasets, including transcriptome and proteome data [25-27]. BN analysis, in this study, was used to predict causal relationships among reactions and subsequently their metabolic modules based on their metabolic fluxes.

With these tools, we herein conceptualized a *f*ramework for network *m*odularization and *B*ayesian network analysis (FMB) in order to investigate the effects of specific perturbation on metabolism and to explore other complementary features of the perturbed cell in the context of metabolism, which other currently available high-throughput techniques do not provide. This framework analyzes a biological system subjected to the specific perturbation by clustering reactions of similar functions (or metabolic flux variation pattern), and graphically displaying direction of influences among these clusters as a result of the specific perturbation. For this, FMB employs constraints-based flux analysis, hierarchical clustering and BN analysis in sequence. For a demonstration, FMB was applied to the genetically perturbed *Escherichia coli* metabolism using its genome-scale metabolic network model [28]. The final outcome is a BN that is a causal network that shows influential correlations among reactions and their metabolic modules that are critical to the metabolic adjustment in response to the specific perturbation.

## Results and discussion

**Figure 1:**
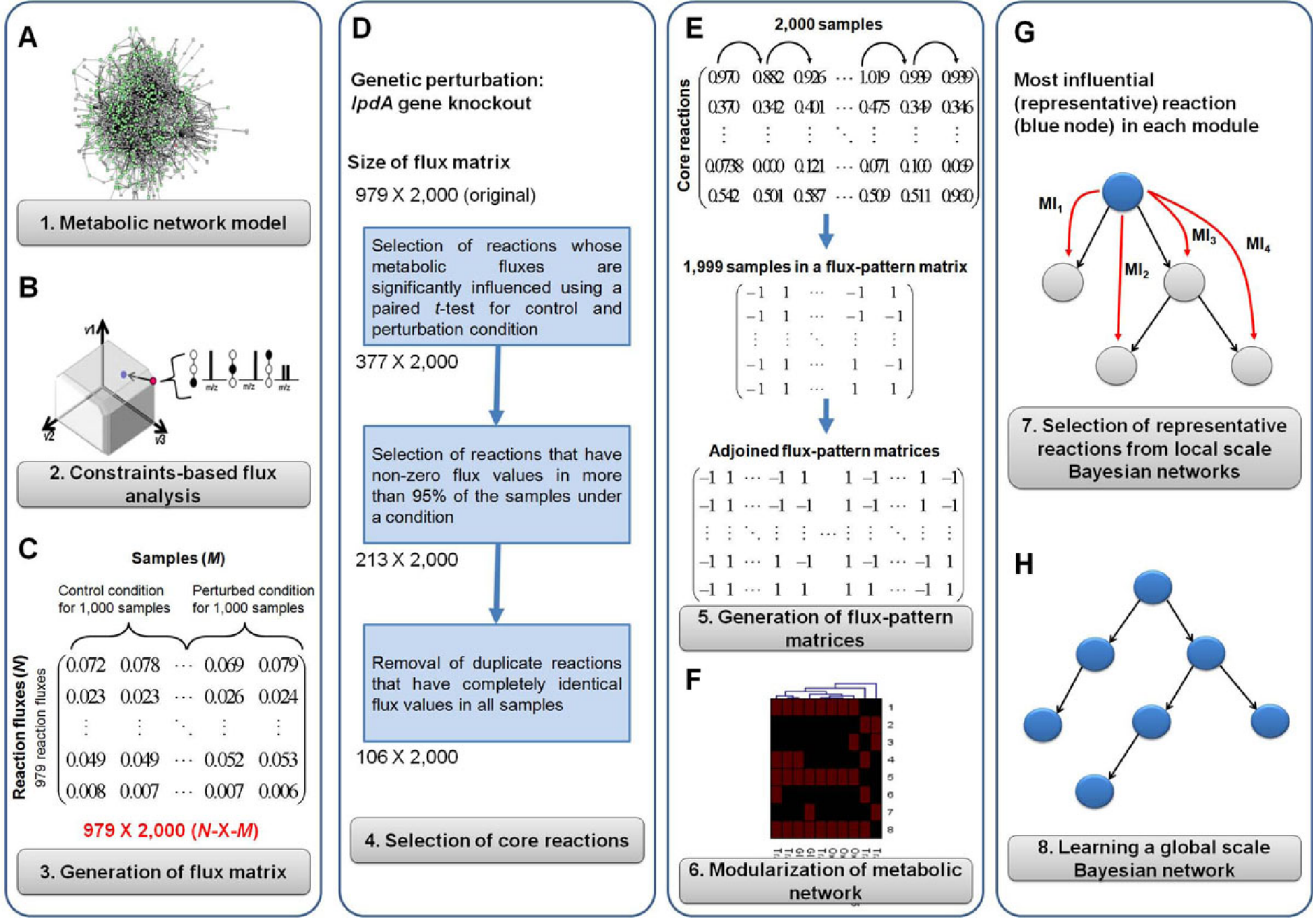
**Schematic procedure of the framework for network modularization and Bayesian network analysis (FMB).** (A and B) Metabolic network model is repeatedly simulated for control and perturbation conditions using constraints-based flux analysis with constraints from ^13^C-based metabolic flux analysis and cell culture to calculate more reliable metabolic flux distributions while amplifying the data size. (C) The result is a flux matrix (*N*-by-*M*) that contains a total of 2,000 samples for each reaction from both control and perturbed condition. (D) Core reactions (see main text for definition) are selected in this step. (E) Flux matrix is converted to flux-pattern matrices that contain information on flux variation pattern from sample to sample, having one of ‘1’, ‘-1’ and ‘0’ (see Methods for details). All the generated flux-pattern matrices are adjoined into a single large flux-pattern matrix for clustering. (F) Hierarchical clustering is applied to this matrix, and reactions are clustered in terms of the uniform functionality, creating metabolic clusters. (G) Bayesian network (BN) of each metabolic module is first inferred, producing local scale BNs, and representative reactions, the most influential ones in their corresponding metabolic module, are determined by measuring the degree of influence of each reaction on others in a module using total mutual information (TMI). Mutual information (MI) is calculated between a target node (blue color) and the other remaining nodes pair by pair, indicated as MI_1_, MI_2_, MI_3_ and MI_4_, in a local scale BN of metabolic module. TMI of the reaction is a summation of these MI values. This procedure is repeated until TMIs of all the other reactions are calculated. At the end, reaction with the highest value of TMI is selected as the representative reaction of the metabolic module. (H) Representative reactions are finally subjected to BN analysis to infer a global scale BN for detailed analysis of specific perturbation given to the biological system.

FMB was utilized in this study to systematically assess the effects of specific perturbation on metabolism at global scale (Figure 1). The goal is to learn causal relationships among clusters of metabolic fluxes in response to a specific perturbation. Here, considerations are that the BN analysis requires many samples or observations of the system of interest, and the number of events or nodes that can be handled by BN analysis is limited, usually to the order of tens [25]. Hence, given a genome-scale metabolic network model (Figure 1A), many samples of genome-scale metabolic flux data need to be generated under the condition of interest, by introducing random errors to each flux value (Figure 1B-C). In this framework, metabolic flux data obtained from wild-type (control condition) and its genetic mutant (perturbed condition) were treated together (see step C below for the reason). Then, the number of reactions was gradually reduced to the level suitable for BN analysis; for this, so called *core reactions* were selected, which are most influenced by specific perturbation, and yet almost always active in the observed samples of either control or perturbed condition [15] (Figure 1D). Next, the metabolic network was modularized based on flux variation pattern of core reactions in order to group functionally correlated reactions (Figure 1E-F). In so doing, reactions clustered in a module were treated as a functional unit. Finally, selecting *representative reaction* from each module, which is the most influential one in the module, leaves adequate number of reactions that can be subjected to BN (Figure 1G-H). Each of these steps is considered in detail as follows.

### Step A-C: Generation of many samples of genome-scale metabolic flux data

Generation of genome-scale metabolic flux data in the first step of the FMB aims at two purposes: the first is to calculate reliable genome-scale metabolic fluxes under specific conditions, and the other is to get a large number of data samples for statistically reliable conclusions through BN analysis. First, fluxes for the branching reactions were constrained with metabolic flux data from ^13^C-based metabolic flux analysis (MFA) [20] in order to obtain reliable intracellular metabolic flux distribution under specific conditions; metabolic fluxes predicted from ^13^C-based MFA are considered to be more reliable than those purely predicted by constraints-based flux analysis. Reactions constrained with experimental data are listed in Table 1 and Methods.

**Table 1:** Physiological constraints used in constraints-based flux analysis for simulating metabolism of wild-type and *lpdA* knockout mutant of *E. coli*

Enzyme	Metabolism	Condition	***v***^^13^ *C*^**or *v***^*fmt*^ (mmol/g dry cell weight/h)	σ
Phosphotransferase system for D-glucose transport	Transport	Wild-type	-3.04	0.01824
		Δ*lpdA* mutant	-2.48	0.1488

Cell growth rate	-	Wild-type	0.20	0.0100
		Δ*lpdA* mutant	0.22	0.0110

Glucose-6-phosphate isomerase	Glycolysis/Gluconeogenesis	Wild-type	2.39	0.3107
		Δ*lpdA* mutant	1.79	0.2327

Pyruvate kinase	Glycolysis/Gluconeogenesis	Wild-type	1.09	0.0109
		Δ*lpdA* mutant	0.26	0.0026

Glucose 6-phosphate dehydrogenase	Pentose phosphate pathway	Wild-type	0.61	0.0732
		Δ*lpdA* mutant	0.64	0.0768

Phosphogluconate dehydrogenase	Pentose phosphate pathway	Wild-type	0.61	0.0732
		Δ*lpdA* mutant	0.32	0.0384

Phosphoenolpyruvate carboxylase	Anaplerotic reactions	Wild-type	0.67	0.0469
		Δ*lpdA* mutant	1.61	0.1127

Phosphoenolpyruvate carboxykinase	Anaplerotic reactions	Wild-type	0.07	0.0070
		Δ*lpdA* mutant	0.93	0.0930

Pyruvate dehydrogenase	Glycolysis/Gluconeogenesis	Wild-type	3.56	0.2136
		Δ*lpdA* mutant	0.00	0.0000

α-ketoglutarate dehydrogenase	Citrate Cycle (TCA)	Wild-type	Not constrained	Not constrained
		Δ*lpdA* mutant	0.00	0.0000

glycine cleavage system	Folate Metabolism	Wild-type	Not constrained	Not constrained
		Δ*lpdA* mutant	0.00	0.0000

Mean values (*v*^^13^*C*^ or *v^fmt^*) of physiological constraints are based on ^13^C-based metabolic flux and cell culture data for the wild-type and *lpdA* knockout mutant of *E. coli*, both grown in continuous culture at dilution rate 0.2 h^-1^[33]. Standard deviations (*σ*) were calculated by multiplying these mean values with average error percentage associated with each reaction, taken from [43]. In addition to these, flux values of α-ketoglutarate dehydrogenase and glycine cleavage system were additionally constrained to zero due to the knockout of *lpdA* gene.

Subsequently, randomly generated errors were introduced to the ^13^C-based metabolic flux and cell culture data constraining the reactions, and constraints-based flux analysis was iteratively performed with these randomly adjusted experimental constraints in order to generate a large number of data samples; as a result, constraints-based flux analysis produces a different solution every time it is performed, satisfying the requirement that statistically reliable BNs demand many observations of the target system (see Methods). Infeasibility of optimization problem from applying too many constraints was handled with least absolute deviation method (see Methods). In this study, a total of 2,000 samples were generated at the end, each 1,000 sets of data from the wild-type (control condition) and its genetic mutant (perturbed condition), respectively. The resulting matrix, *N*-by-*M*, is named *flux matrix* in this study, where *N* is the number of reactions and *M* is the number of samples; *M* is 2,000 in this study (Figure 1C). It is important to note that flux data samples generated for the two different conditions are combined into a single flux matrix in order to reflect the effects of perturbation on the metabolic flux distribution, which enables BN analysis to more reliably infer the directionality of causal relationships among reactions and their modules [26].

### Step D: Selection of core reactions

In the flux matrix generated, the number of reactions needs to be reduced to the size that can be handled by BN analysis because only a fraction of reactions, namely metabolic core reactions, govern the metabolic activity [15], and the network model has many functionally duplicating reactions that show exactly the same metabolic flux values under all circumstances, and do not contribute to additional information. For this, a series of reduction processes were performed to select only the core reactions from the flux matrix (Figure 1D). The definition of core reaction in this study includes 1) a feature of significant flux variations accompanied by specific perturbation and 2) being almost always active under all samples of either control or perturbation condition [15]. Here, *being almost always active* refers to having non-zero fluxes for a reaction in more than 95% of samples under either control or perturbed condition, thereby becoming a core reaction. This shows a slight contrast with the previous definition that the core reactions be always active under all circumstances [15]. This new definition was to sufficiently cover the number of reactions suitable for BN analysis.

With this in mind, reactions were first selected, which were highly influenced by perturbation in terms of variation in flux values using a paired *t*-test; two sets of 1,000 flux values from control and perturbation condition were compared for each reaction. Reactions with P < 10^-2^ were considered to be significantly influenced by the perturbation. Among significantly influenced reactions, reactions that have non-zero fluxes in more than 95% of samples under control or perturbed condition were selected, based on the aforementioned definition of core reaction, by which they coordinate and significantly contribute to the metabolic integrity [15]. Next, for *duplicate reactions*, reactions that have exactly the same flux values in all samples, only one of them was selected because they do not contribute to the robust statistical conclusion, but only increase the data volume (Additional file 1). The removed duplicate reactions were considered in the later stage of data analysis after all statistical calculations. The final remaining reactions, core reactions, are the major fluxes operating the cell metabolism, and significantly influenced by given perturbation (Additional file 1). Core reactions were then subjected to subsequent modularization and BN analysis.

### Step E and F: Modularization of metabolic network based on flux variation pattern

Metabolism was then modularized by grouping core reactions with metabolically correlated functions using hierarchical clustering; this modularization enables us to look at modules of reactions as functional unities instead of individual reactions, thereby simplifying our subsequent analysis (Additional file 1). Here, flux variation was used as a criterion for clustering, such that reactions that have similar pattern of flux variation from sample to sample were grouped. This is analogous to clustering of genes based on their expression level in transcriptome data [29]. Specifically, the *reduced flux matrix* containing only the core reactions was converted to a *flux-pattern matrix* that represents the flux variation pattern from one sample to another (Figure 1E-F) (see Methods). As a result, reactions in a cluster show synchronized patterns of increases (designated by the value ‘1’ in the flux-pattern matrix) and decreases (designated by ‘-1’ in the flux-pattern matrix) in flux values in response to the perturbation, implying that they pursue correlative biological functions (see Methods for further details on generation of various flux-pattern matrices and their adjoined form). Be noted that the term *cluster* and *module* are interchangeably used to discuss the results henceforth.

### Step G and H: Bayesian network analysis of metabolic modules

Finally, BN analysis was employed to predict causal relationships of reactions at local and global scale. At local scale, BN of reactions in a cluster was first inferred (Additional file 2), and representative reactions that most heavily influence other reactions in each metabolic cluster were identified by using the concept of mutual information (MI) [30] and total mutual information (TMI) [31,32]; representative reactions have the highest value of TMI, and this procedure is detailed in Methods. Selected representative reactions from each module were then subjected to another round of BN analysis, yielding a *global scale BN* (Figure 1G-H; Additional file 1). Because reactions in each module possess positively correlated functional features, or flux variation pattern, we speculate that BN with representative reactions would reveal a reasonable system-wide causal network that shows correlations among their corresponding metabolic modules as well in response to specific perturbations.

Although the FMB looks into correlations that exist in metabolism system, it is distinct from previously established relevant approaches, for instance flux coupling analysis [12], and uniform random sampling and subsequent calculation of correlation coefficients among reactions [14] in several aspects: (1) FMB reveals correlations among metabolic modules in addition to individual reactions by considering flux variation patterns of reactions using clustering, thereby showing modular-level behaviours under specific condition; (2) causal relationships predicted from the FMB are graphically shown with arrows, which provides better readability; (3) FMB is more oriented to context-specific biological issues, such as specific gene knockout, by imposing constraints of mutant-specific ^13^C-based metabolic flux and cell culture data. These distinctions of FMB would provide complementary aspects of the cell that the previously reported approaches do not provide.

### Application of FMB to *lpdA* gene knockout mutant of *E. coli*

As a demonstration of the framework FMB, it was applied to the wild-type and *lpdA* mutant of *E. coli* to systematically evaluate how the genetic perturbation, *lpdA* gene knockout, affects *E. coli* metabolism, cultured in defined minimal media with glucose [33]. This *lpdA* gene encodes lipoamide dehydrogenase, which is an important component of pyruvate dehydrogenase complex, α-ketoglutarate dehydrogenase, and glycine cleavage system [34,35]. Therefore, LpdA assumes an important biological role in metabolism at broad scope as a component of enzyme complexes: pyruvate dehydrogenase complex for connecting glycolysis and TCA cycle, and producing acetyl-CoA and CO_2_ under aerobic condition, α-ketoglutarate dehydrogenase for generating NADH from the operation of TCA cycle, and glycine cleavage system for one carbon metabolism that is associated with nucleotides and cofactors [33]. Consequently, such biological importance and global effects that LpdA is likely to exert on the cellular physiology made this enzyme component an ideal target for the application of FMB. Results of each step in FMB are represented as follows.

#### Step A-C: Generation of flux matrix from metabolic network model

Flux matrix containing 979 reactions and 2,000 data samples were first generated from constraints-based flux analysis with constraints of ^13^C-based metabolic flux and cell culture data under control and perturbation conditions, combined with random errors (Figure 1A-C). It should be noted that flux values from *lpdA* knockout mutant were obtained by additionally constraining the flux values of pyruvate dehydrogenase complex, α-ketoglutarate dehydrogenase, and glycine cleavage system to zero in order to reflect the knockout of *lpdA* gene.

#### Step D: Selection of core reactions

Flux matrix was then subjected to a series of filtering processes; initial 979 reactions were reduced to 377 reactions from *t*-test, 213 reactions after filtering reactions that have non-zero fluxes in more than 95% of samples in either conditions, and finally 106 core reactions after removing duplicate reactions and selecting one of them for subsequent analysis (Figure 1D; Additional file 1).

#### Step E and F: Modularization of metabolic network from flux-pattern matrices

The reduced flux matrix was next converted to flux-pattern matrices (Figure 1E), and hierarchical clustering was performed (Figure 1F). A total of 44 clusters, or metabolic modules, were created with size of each cluster ranging from 1 to 30 reactions (Additional file 1 and 2). Duplicate reactions that were previously filtered in the flux matrix were also considered at this stage by inserting all duplicate reactions in the same cluster. Overall, reactions involved in the same submetabolism were grouped into the same cluster, partially demonstrating that flux variation pattern employed as a criterion for hierarchical clustering does well represent the functional aspect of reactions. Cluster 5 (cell envelope biosynthesis), cluster 11 (tyrosine, tryptophan, and phenylalanine metabolism), and cluster 15 (membrane lipid metabolism) are the exemplary clusters that have reactions from the same submetabolism. For clusters having reactions from different submetabolisms, biochemical link that connects these reactions together can be inferred. Cluster 28 belongs to this case; reactions from TCA cycle, such as succinate dehydrogenase, and those from oxidative phosphorylation, including cytochrome oxidase and ATP synthase, as well as oxygen transporter are clustered in the same module as they all are linked through aerobic electron transfer for respiration. Likewise, reactions from cell envelope biosynthesis and membrane lipid metabolism tend to be clustered together because fatty acids created from membrane lipid metabolism are fed into the biosynthesis of cell envelope. Cluster 42 is such a case, and has, in addition, arabinose-5-phosphate isomerase that produces arabinose-5-phosphate critical for lipopolysaccharide and alanine racemase that converts L-alanine to D-alanine, an essential building block of peptidoglycan. All these examples so far discussed indicate that reactions can be grouped together based on their functional characteristic that is flux variation pattern from sample to sample. Because each cluster represents functionally similar reactions, this modularization process will ease our subsequent interpretation of the data.

#### Step G and H: Generation of a global scale Bayesian network using representative reactions

Once the *E. coli* metabolism was modularized, representative reaction for each cluster was selected (Figure 1G), and BN analysis was applied to them in order to investigate the causal relationships existing among these clusters at global scale (Figure 1H; Additional file 1). Because each module is presented as a representative reaction in the global scale BN, other reactions in their corresponding cluster should also be considered during interpretation. Good starting point for the detailed interpretation would be nodes that contain reactions directly perturbed, genetically or environmentally, or those that have the highest number of links influencing others.

### Analysis of the results from FMB

**Figure 2:**
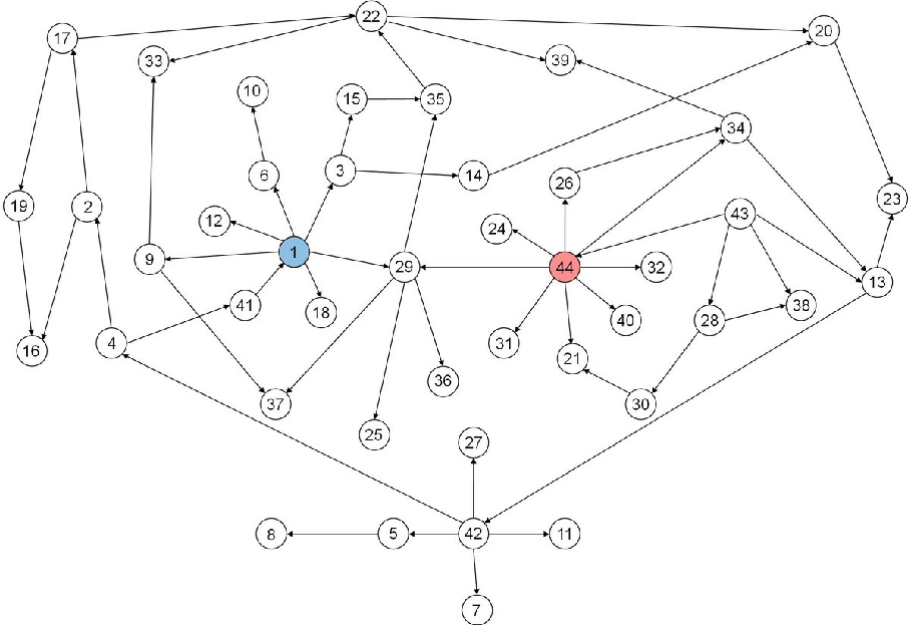
**Global scale Bayesian network from FMB applied to wild-type and *lpdA* gene knockout mutant of *E. coli*.** Global scale Bayesian network was inferred from metabolic flux data on wild-type and *lpdA* mutant of *E. coli*. Number in each node indicates the cluster number (see Additional file 1 for details). Cluster 44 in red contains a reaction directly perturbed by genetic perturbation, and influences the largest number of nearby reactions. Cluster 1 in blue influences the second largest number of reactions.

**Figure 3:**
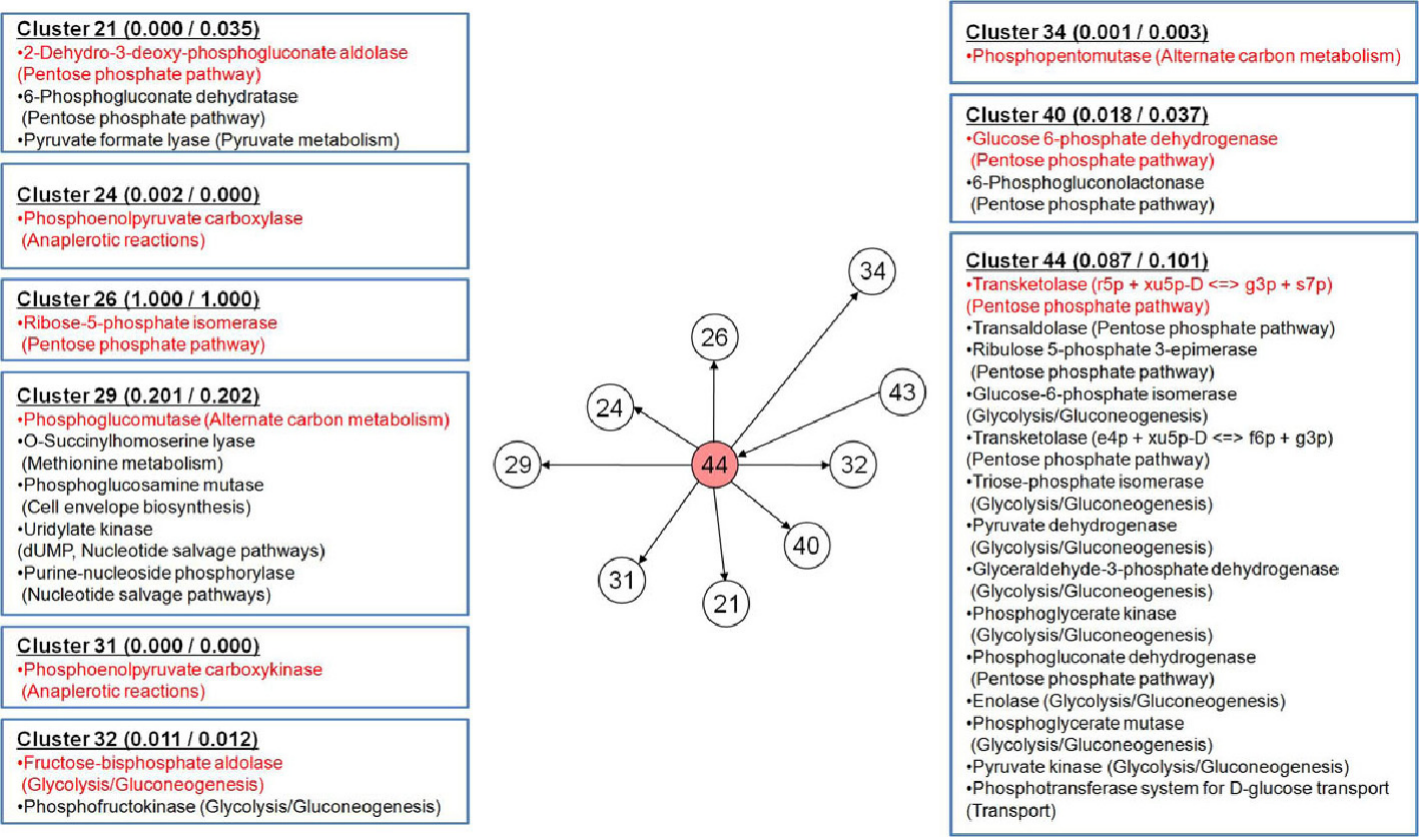
**Reactions constituting cluster 44 and nearby clusters from wild-type and *lpdA* mutant of *E. coli*.** Clusters 21, 24, 26, 29, 31, 32, 34 and 40 are under direct influence of cluster 44. Each box contains information on reactions in the order of their respective TMI values for each cluster (see Additional file 1 for details). Numbers next to each cluster number are the calculated average essentiality of reactions in the cluster for the wild-type and *lpdA* mutant in order; the higher the essentiality value is, the more essential the reaction is. Reactions in red are the representative reactions. Parenthesis next to the enzyme name shows the name of submetabolism, in which the enzyme is involved. To differentiate two transketolases in cluster 44, their corresponding reactions are shown next to the enzyme name. Cluster 43 influencing cluster 44 contains three reactions: 4-aminobutyrate transaminase (arginine and proline Metabolism), glutamate decarboxylase (glutamate metabolism), and isocitrate dehydrogenase (TCA cycle). Abbreviations are: e4p, D-erythrose 4-phosphate; r5p, alpha-D-ribose 5-phosphate; f6p, D-fructose 6-phosphate; g3p, glyceraldehyde 3-phosphate; s7p, sedoheptulose 7-phosphate; xu5p-D, D-xylulose 5-phosphate.

The resulting global scale BN of representative reactions from the metabolic flux data of wild-type and *lpdA* mutant of *E. coli* is shown in Figure 2. Of our primary interest is cluster 44 that contains pyruvate dehydrogenase whose activity is directly disrupted by removal of *lpdA* gene (Figure 3). Cluster 44 consists of reactions associated with glycolysis/gluconeogenesis, pentose phosphate pathway and transport of glucose via phosphotransferase system, and has transketolase-catalyzed reaction as a representative reaction (Figure 3). This cluster was predicted to directly influence following metabolic clusters: cluster 21 (2-dehydro-3-deoxy-phosphogluconate aldolase, 6-phosphogluconate dehydratase and pyruvate formate lyase), 24 (phosphoenolpyruvate (PEP) carboxylase), 26 (ribose-5-phosphate isomerase), 29 (phosphoglucomutase, O-succinylhomoserine lyase, phosphoglucosamine mutase, uridylate kinase and purine-nucleoside phosphorylase), 31 (PEP carboxykinase), 32 (fructose-bisphosphate aldolase and phosphofructokinase), 34 (phosphopentomutase) and 40 (glucose 6-phosphate dehydrogenase and 6-phosphogluconolactonase) (Figure 3). Size of these clusters is relatively small, and their reactions are mainly associated with central carbon metabolism with exception of cluster 29. This influential network is biologically reasonable as most of reactions in these clusters are connected through PEP and pyruvate or within their proximity. Hence, genetic perturbation of *lpdA* redistributes the fluxes of its nearby reactions in central carbon metabolism through cluster 44.

**Figure 4:**
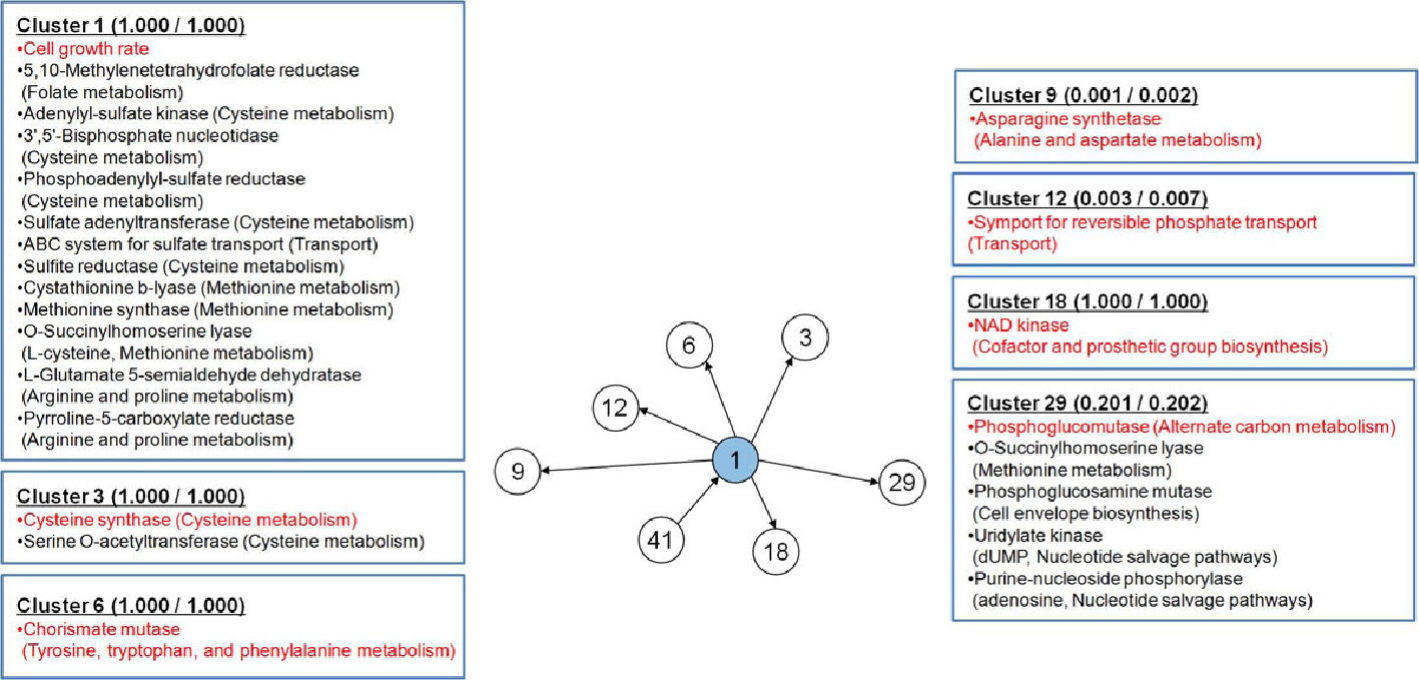
**Reactions constituting cluster 1 and nearby clusters from wild-type and *lpdA* mutant of *E. coli*.** Clusters 3, 6, 9, 12, 18 and 29 are under direct influence of cluster 1. Each box contains information on reactions in the order of their respective TMI values for each cluster (see Additional file 1 for details). Numbers next to each cluster number are the calculated average essentiality of reactions in the cluster for the wild-type and *lpdA* mutant in order; the higher the essentiality value is, the more essential the reaction is. Reactions in red are the representative reactions. Parenthesis next to the enzyme name shows the name of submetabolism, in which the enzyme is involved, and substrates for O-succinylhomoserine lyase (cluster 1, L-cysteine), uridylate kinase (cluster 29, dUMP) and purine-nucleoside phosphorylase (cluster 29, adenosine). Cluster 41 influencing cluster 1 contains 14 reactions, associated with: glycolysis/gluconeogenesis, histidine metabolism, membrane lipid metabolism, and tyrosine, tryptophan, and phenylalanine metabolism. Its representative reactions are prephenate dehydrogenase and tyrosine transaminase, both involved in tyrosine, tryptophan, and phenylalanine metabolism.

Metabolic cluster 1 that has a reaction for cell growth rate as a representative reaction was also shown to influence high number of other clusters: cluster 3 (cysteine synthase and serine O-acetyltransferase), 6 (chorismate mutase), 9 (asparagine synthetase), 12 (phosphate transporter), 18 (NAD kinase) and 29 (phosphoglucomutase, O-succinylhomoserine lyase, phosphoglucosamine mutase, uridylate kinase and purine-nucleoside phosphorylase) (Figure 4). Unlike cluster 44, cluster 1 influences metabolically wider range of reactions, mostly related with amino acid biosynthesis (Figure 4). Cluster 1 itself is also composed of many amino acid-metabolic reactions. These evidences manifest that amino acid biosynthesis is a major submetabolism affected by changes in cell growth rate. This becomes obvious, considering the fact that protein constitutes more than half of total *E. coli* dry cellular weight [36]. Appearance of transporter for sulfate in cluster 1 and phosphate in cluster 12 is also notable as biosynthesis of some amino acids requires these two inorganic molecules.

As cell growth rate is reduced upon knockout of *lpdA *[33], it is noteworthy to observe how the cluster 1 and 44, which contain reactions of cell growth rate and pyruvate dehydrogenase, respectively, are correlated in the global scale BN (Figure 2). Although these two clusters were both shown to influence cluster 29, and there is no direct influential path from cluster 44 to 1 or vice versa, we presume that reduction of the cell growth rate and fluxes of other functionally coordinated reactions in the cluster 1 were initially caused by the negatively affected reactions of cluster 29. This causal relationship becomes clearer when we consider the fact that phosphoglucomutase is the representative reaction of cluster 29, which converts glucose-6-phosphate to glucose-1-phosphate that is a precursor of glycogen and cell envelope, and highly associated with biomass formation. It was reported that excessive accumulation of glycogen in *E. coli* resulted in increased biomass formation, which, in some part, experimentally supports the correlation between cluster 1 and 29 [37]. In turn, cluster 29 seems to have been affected by cluster 44 as inferred in the global scale BN because *lpdA* knockout mutant cannot efficiently generate energy for biosynthesis of biomass components, compared to the wild-type, due to the blocked pyruvate dehydrogenase that hinders further oxidation of glucose for oxidative phosphorylation (Figure 3). Here, it should be noted that, along with glycogen, enzymes involved in rather wider range of metabolisms, including amino acids, cell envelope, and nucleotides in cluster 29, indicate that various spots in the metabolic network are likely to be influenced by the removal of *lpdA*, which, in turn, might have negatively affected the cell growth rate. As a result, the global scale BN reveals closer positioning of cluster 1 and 44 through cluster 29.

Finally, essentiality of reactions calculated with constraints-based flux analysis provided additional complementary information on the clusters; essentiality has a scale of 0 to 1, and reactions with greater essentiality receives the value closer to 1 (Methods). First, reactions involved in clusters 1 to 44 had average essentiality of 0.744 and 0.748 for the wild-type and *lpdA* knockout mutant, respectively, while average essentiality of whole reactions was 0.215 and 0.216 under respective condition. This indicates that reactions collected into the clusters are heavily associated with cellular energy production, in comparison with those not included in the clusters. Closer examination of these clustered reactions reveals that clusters surrounding cluster 44 have relatively low essentiality (Figure 3), indicating that they are likely to be more committed to flux redistribution upon *lpdA* knockout than creating essential biomass constituents. This observation is consistent with the previous report, in that a set of reactions responsible for carbon flux distribution in *E. coli* are consistently included in cluster 44 and its surrounding clusters [38]. On the contrary, cluster 1 has higher number of surrounding clusters that have complete essentiality (= 1.000), which shows that they are more directly associated with biomass formation (Figure 4). Interestingly, although reactions in these 44 clusters cover most of the reactions in the *metabolic core* previously reported in Almaas *et al.*, which are defined to be always active under all examined circumstances [15], most of those overlapping reactions were gathered in the clusters that are not under direct influence of the cluster 1 and 44. From this, it can be considered that FMB in this study identifies reactions that are under direct influence of the specific perturbation, *lpdA* knockout in this case, in addition to the reactions required for the survival of the organism, which corresponds to the metabolic core [15].

Taken together, FMB provides theoretical evidences for the possible correlations among metabolic modules in response to the perturbation, as exemplified by cluster 1, 29, and 44 in this study. This study would consequently reveal additional pictures of the cellular physiology that could complement conventionally available biological information.

## Conclusions

FMB developed in this study groups functionally similar reactions, and subsequently investigates the causal patterns among metabolic clusters in response to specific perturbation. This newly generated knowledge of causal patterns enables us to capture significant changes in metabolism at the level of metabolic modules, which distinguishes itself from conventional MFA. Hence, it could be used as another tool for examining the microbial physiology in addition to currently available genome-wide high-throughput techniques [39]. Furthermore, this FMB could be applied to interpreting different types of specific perturbations, including multiple gene knockouts and environmental stresses.

## Methods

Overall scheme and rationale for each step of FMB are elaborated in Results and Discussion (Figure 1) while its critical computations steps that require extensive explanation are mentioned in this section. All the computations were performed using 2.80 GHz Intel i5 processors.

### Genome-scale metabolic network model of *E. coli*

In this study, a previously reported genome-scale metabolic network model of *E. coli* was used [28]. This model is comprised of 979 reactions and 814 metabolites. Simulations were performed using the program package MetaFluxNet [40] and GAMS (GAMS Development Corp., Washington DC, USA) for the optimization technique developed in this study as detailed below.

### Simulation of the metabolic network model with random generation of constraints based on ^13^C-based metabolic flux data

Simulation of the metabolic network model along with data preprocessing were implemented in GAMS for the wild-type (control condition) and *lpdA* mutant (perturbed condition), separately. For application of FMB to *E. coli* metabolism, ^13^C-based metabolic flux data were adopted from Li *et al. *[33] for wild-type (control) and *lpdA* mutant (perturbation) for genetic perturbation. All constraints were from the continuous culture of *E. coli* at dilution rate of 0.2 h^-1^ under the defined minimal medium with glucose (Table 1). Here, instead of considering all the reactions, only those heavily responsible for the split of fluxes between major metabolic pathways were constrained with the ^13^C-based metabolic flux data (Table 1); they include glucose-6-phosphate isomerase, pyruvate kinase, glucose 6-phosphate dehydrogenase, phosphogluconate dehydrogenase, PEP carboxylase, PEP carboxykinase and pyruvate dehydrogenase [38]. In addition, phosphotransferase system for glucose uptake and cell growth rate were also given constraints [33]. Metabolic fluxes of these constrained reactions were allowed to be reconciled within the range of their standard deviation (Table 1). In addition to pyruvate dehydrogenase above, flux values of α-ketoglutarate dehydrogenase and glycine cleavage system were also additionally constrained to zero for calculating metabolic flux data for *lpdA* gene knockout mutant.

The simulation by optimization often produces infeasible solutions when a large number of constraints are used [41]. There certainly exist gaps between flux values from constraints-based flux analysis and experimental data, such that flux data need to be reconciled. To overcome this infeasibility, we implemented least absolute deviation [42]. The objective function was to minimize the distance between theoretical (*v^con^*) and experimental fluxes with randomly assigned errors (*v_k_* and *v_b_*), namely ^13^C-based metabolic flux and cell culture data, for the reactions catalyzed by the aforementioned enzymes, such that metabolic fluxes were reconciled to give realistic metabolic flux distributions. It should be noted that random errors are required to be within the standard error of the experimental measurements. This whole procedure is mathematically formulated as follows:

where *S* is a stoichiometric matrix that consists of metabolite *i* in reaction *j* (*i* x *j*), and *v* is a vector showing the flux of reaction *j* (*j* x 1). α and β are lower and upper bounds for the flux of reaction *j*. *K*, a subset of *J*, is a set of reactions constrained with ^13^C-based metabolic flux data, and *B*, the other subset of *J*, is a set of reactions constrained with the rates of cell growth and metabolite secretion from the continuous cell culture. *v^con^* is a vector of fluxes calculated from constraints-based flux analysis whose experimental values are available. *v^13C^* is a vector of intracellular fluxes obtained from ^13^C-based MFA, and *v^fmt^* is a vector of the cell growth rate and glucose uptake rate from the continuous cell culture. *v_k_* and *v_b_* are constrained to be randomized values, being equivalent to *v^13C^* and *v^fmt^* with randomly added errors, respectively. Finally, *random*(*μ*,*σ*) is a function that generates a random number according to normal distribution with mean *μ* and standard deviation *σ*. In this study, mean was set to the flux value from ^13^C-based MFA, *v^13C^*, or the rates of cell growth and glucose uptake, *v^fmt^*, and standard deviation was differently set for each reaction according to the measurement error reported in the literature [43]. Above optimization formulation was iterated 1,000 times using linear programming for each wild-type (control condition) and *lpdA* mutant (perturbed condition), yielding a total of 2,000 sets of data. Flux data generated for both conditions were merged into a flux matrix, so that the directionality of causal relationships can properly be inferred by BN analysis [26].

### Modularization of metabolic network based on flux variation pattern

For conversion of reduced flux matrix having only core reactions into flux-pattern matrix that contains information on the flux variation patterns, flux value of a reaction sample (*m^th^* column) in the reduced flux matrix was compared to that of its following sample (*m*+*1^th^* column), and ‘1’ is given in the position (*n*, *m*) of flux-pattern matrix if the flux value of the reaction in (*n*, *m*) is increased in (*n*, *m*+*1*) of the flux matrix. ‘-1’ is given if the flux value is decreased in the following sample, and ‘0’ is given for the same flux values in the *m^th^* and *m*+*1^th^* sample (Figure 1E). This was done for all the reactions in the reduced flux matrix, completing a flux-pattern matrix. Therefore, the flux-pattern matrix uses ‘-1’, ‘0’ or ‘1’ to represent the flux variation pattern of a reaction from sample to its following sample. Here, the flux-pattern matrix may be different, depending on the order of columns (samples) in the reduced flux matrix. Therefore, columns of the reduced flux matrix were randomly permutated, and the flux-pattern matrix was obtained accordingly (Figure 1E). Then, flux-pattern matrices independently generated from flux matrices with different order of samples (columns) were adjoined and subjected to hierarchical clustering. In this study, we tested adjoined matrices with different number of constituting individual flux-pattern matrices for hierarchical clustering in order to confirm that they produce consistent clusters of metabolic reactions. Adjoined 300 flux-pattern matrices showed agreeable consistency every time they were produced from metabolic flux data. Finally, it should be noted that flux-pattern matrices consisting of ‘-1’, ‘0’ and ‘1’ were used only for clustering process. Once metabolic clusters were formed, previously calculated metabolic flux values (step A to C of FMB) corresponding to each clustered reaction were used in the subsequent BN analysis.

Conversion of reduced flux matrices with permutated samples (columns) into flux-pattern matrices, adjoining flux-pattern matrices and hierarchical clustering were conducted in Matlab (Mathworks). For hierarchical clustering, the “clusterdata” command was used. Euclidean distance, average linkage and cutoff value of 1.0 were used as calculation options.

### Bayesian network analysis

BN is a probabilistic graphical model, which represents the causal relationships among variables, and it is important that its graph be acyclic. Nodes in the probabilistic graphical model correspond to random variables, which represent the fluxes of biochemical reactions in metabolic network, and its directed arcs from a variable to another indicate the direction of influences between them. In particular, node at tail of the directed arc is called “parent” and node at head of the arc is called “descendent.” Such statistical dependence is characterized by Markov condition, which states that each variable *X_i_* is conditionally independent of the set of all its non-descendents, given its parents *Pa_i_* in the graph [24]. Based on this Markov condition, the multivariate joint probability distribution of the graphical model can be expressed as follows:

One of the goals of this study is to infer such BNs from metabolic flux profiles. All the BN analyses were performed with commercially available software BayesiaLab (BAYESIA, Laval, France) [44,45]. Firstly, a conditional probability table of all the nodes needs to be defined in order to infer BNs. Therefore, flux values need to be discretized into discrete variables in terms of probability. In BayesiaLab, equal frequency method was employed for this purpose, which preprocessed the flux values in 2,000 sets of metabolic flux data from control and perturbed conditions. This method divides the range of continuous attribute into intervals (four intervals in this study) where each interval contains equal or similar number of data points (i.e. flux values). Structure learning of BNs was then performed with tabu order algorithm for generating various candidate networks along with minimum description length (MDL) as a scoring criterion [46]. Structure learning is finding the network that best describes the causal relationships in the data, evaluated by specific scoring criterion. Tabu order algorithm searches the solution space of all possible candidate networks by adding, deleting or reversing arcs based on its specific rules. MDL then evaluates each BN with respect to the data, and selects the most likely one that describes the data. Here, MDL accounts for the trade-off between accuracy and complexity of the learnt BN, and thus the optimal BN is both simple and accurate model of the data [46].

### Identification of representative reactions in each metabolic module

BN analysis and MI were employed to identify a representative reaction for each metabolic module. BN analysis was first applied to reactions in each module, so that BN of each module was inferred, namely local scale BN. MI was then employed to assess the degree of influence that each reaction exerts on other reactions. MI is a concept adopted in information theory, which calculates the mutual dependence between two random variables [30]. The higher the MI value is, the more likely the two variables are correlated. MI *I*(*x*; *y*) is represented as:

*p*(*x*, *y*) is a joint probability mass function, and *p*(*x*) and *p*(*y*) are marginal probability mass functions. Let *y* be a child node and *x* be its parent node. Then, the MI between *x* and *y* describes the mutual influence between the two. Specifically for this work, we extended the concept of MI to TMI [31,32] to measure the impact of a reaction on other remaining reactions in the module (Figure 1G) [31]. To identify a representative reaction using TMI in each metabolic cluster, a metabolic reaction was first set as a target node, and the MIs between this target node and others were calculated one by one as a pair (Figure 1G). All these MI values were summed up to indicate the relative weight or influence of the reaction (i.e. target node) on its corresponding metabolism (i.e. metabolic cluster). TMI is defined as:

*t* is the target node and *r*_1,…,*n*_ are the remaining nodes in the BN. This procedure was repeated by selecting other remaining reactions as the target node and calculating the TMIs likewise. At the end, a reaction with the highest TMI value was identified as the representative reaction. Representative reactions determined from each local scale BN were then subsequently subjected to another BN analysis altogether to infer causal relationships among metabolic fluxes on a global scale. From this procedure, global scale BN is completed, consisting of representative reactions for each metabolic module, thereby explicitly revealing possible influences existing among them.

### Calculation of reaction essentiality

Essentiality for each reaction was obtained by calculating the cell growth rate with that reaction removed using constraints-based flux analysis. The calculated cell growth rate for each reaction was then scaled (= 1 – *g*∆/*g*), wherein *g*∆ and *g* are the calculated cell growth rates of the wild-type and gene knockout mutant, respectively [47]. Essentiality of zero indicates that the removed reaction has no effects on the cell growth rate, while essentiality of one means complete stoppage of the cell growth rate for the removed reaction.

## List of abbreviations

BN: Bayesian network; FMB: framework for network modularization and Bayesian network analysis; MDL: minimum description length; MFA: metabolic flux analysis; MI: mutual information; PEP: phosphoenolpyruvate; TMI: total mutual information.

## Competing interests

The authors declare that they have no competing interests.

## Authors' contributions

TYK and SYL conceived the study and directed the project. HUK implemented the framework, and HUK, TYK and SYL analyzed the data. All authors read and approved the final manuscript.

## Supplementary Material

Additional file 1 **Table S1.** Detailed information of clustered core reactions and their duplicate reactions in *E. coli* metabolism perturbed with *lpdA* knockout. Click here for file

Additional file 2 **Figure S1.** Local scale Bayesian networks of clusters from wild-type and *lpdA* mutant of *E. coli*. Click here for file
