# Supplementary material for: Framework for network modularization and Bayesian network analysis to investigate the perturbed metabolic network
Source: BMC Syst Biol. 2011 Dec 14;5(Suppl 2):S14. doi: 10.1186/1752-0509-5-S2-S14 (PMC3287480; doi:10.1186/1752-0509-5-S2-S14)
Supplement: Additional file 1 — Table S1. Detailed information of clustered core reactions and their duplicate reactions in E. coli metabolism perturbed with lpdA knockout. [file 1752-0509-5-S2-S14-S1.pdf]

Table S1. Detailed information of clustered core reactions and their duplicate reactions in *E. coli* metabolism perturbed with *lpdA* knockout. Information on all the core reactions and their duplicate reactions used for the generation and interpreation of local and global scale BNs is shown. Their enzyme abbreviations are also shown in the column named "iJR904" to facilitate search for their further information in Reed et al. Genome Biol. 4:R54 (2003). Reactions indicated with "b" in the "BN" column are representative reactions of their metablic module, and is used for inference of global scale BN. Reactions indicated with d'x' where x is a integer in "Duplicate" column are duplicate ones; those with the same integer mean that they are the equivalent duplicate reactions and have the same total mutual information (TMI) values. Reactions in each cluster are listed in the decreasing order of TMI values.

Cluster No.	iJR904	Metabolism	TMI	BN	Duplicate	Enzyme
1	BiomassFlux	-	2.1705	b		cell growth rate
1	MTHFR2	Folate Metabolism	2.1553			5,10-methylenetetrahydrofolate reductase (NADH)
1	ADSK	Cysteine Metabolism	2.1501		d1	adenylyl-sulfate kinase
1	BPNT	Cysteine Metabolism			d1	3',5'-bisphosphate nucleotidase
1	PAPSR	Cysteine Metabolism			d1	phosphoadenylyl-sulfate reductase (thioredoxin)
1	SADT2	Cysteine Metabolism			d1	sulfate adenyltransferase
1	SULabc	Transport, Extracellular			d1	sulfate transport via ABC system
1	SULR	Cysteine Metabolism			d1	sulfite reductase (NADPH2)
1	CYSTL	Methionine Metabolism	2.1012		d2	cystathionine b-lyase
1	METS	Methionine Metabolism			d2	methionine synthase
1	SHSL1	Methionine Metabolism			d2	O-succinylhomoserine lyase (L-cysteine)
1	G5SADs	Arginine and Proline Metabolism	2.0996		d3	L-glutamate 5-semialdehyde dehydratase (spontaneous)
1	P5CR	Arginine and Proline Metabolism			d3	pyrroline-5-carboxylate reductase
2	KAS16	Membrane Lipid Metabolism		b		3-hydroxy-myristoyl-ACP synthesis
2	DTMPK	Nucleotide Salvage Pathways			d4	dTMP kinase
2	NDPK4	Nucleotide Salvage Pathways			d4	nucleoside-diphosphate kinase (ATP:dTDP)
3	CYSS	Cysteine Metabolism		b	d5	cysteine synthase
3	SERAT	Cysteine Metabolism			d5	serine O-acetyltransferase
4	C161SN	Membrane Lipid Metabolism		b		fatty acid biosynthesis (n-C16:1)
5	ALAALAr	Cell Envelope Biosynthesis	1.0466	b	d6	D-alanine-D-alanine ligase (reversible)
5	PAPPT3	Cell Envelope Biosynthesis			d6	phospho-N-acetylmuramoyl-pentapeptide-transferase (meso-2,6-diaminopimelate)
5	PPTGS	Cell Envelope Biosynthesis			d6	peptidoglycan subunit synthesis
5	UAAGDS	Cell Envelope Biosynthesis			d6	UDP-N-acetylmuramoyl-L-alanyl-D-glutamyl-meso-2,6-diaminopimelate synthetase
5	UAGCVT	Cell Envelope Biosynthesis			d6	UDP-N-acetylglucosamine 1-carboxyvinyltransferase
5	UAGPT3	Cell Envelope Biosynthesis			d6	UDP-N-acetylglucosamine-N-acetylmuramyl-(pentapeptide)pyrophosphoryl-undecaprenol N-acetylglucosamine transferase
5	UAMAGS	Cell Envelope Biosynthesis			d6	UDP-N-acetylmuramoyl-L-alanyl-D-glutamate synthetase
5	UAMAS	Cell Envelope Biosynthesis			d6	UDP-N-acetylmuramoyl-L-alanine synthetase
5	UAPGR	Cell Envelope Biosynthesis			d6	UDP-N-acetylenolpyruvoylglucosamine reductase
5	UDCPDP	Cell Envelope Biosynthesis			d6	undecaprenyl-diphosphatase
5	UGMDDS	Cell Envelope Biosynthesis			d6	UDP-N-acetylmuramoyl-L-alanyl-D-glutamyl-meso-2,6-diaminopimeloyl-D-alanyl-D-alanine synthetase
5	GLUR	Cell Envelope Biosynthesis			d6	glutamate racemase
5	DAGK_EC	Cell Envelope Biosynthesis	1.0168		d7	diacylglycerol kinase
5	PEPT_EC	Cell Envelope Biosynthesis			d7	ethanolamine phosphotransferase
5	U23GAAT	Cell Envelope Biosynthesis			d7	UDP-3-O-(3-hydroxymyristoyl)glucosamine acyltransferase
5	UAGAAT	Cell Envelope Biosynthesis			d7	UDP-N-acetylglucosamine acyltransferase
5	UHGADA	Cell Envelope Biosynthesis			d7	UDP-3-O-acetylglucosamine deacetylase
5	AGMHE	Cell Envelope Biosynthesis	1.0034		d8	ADP-D-glycero-D-manno-heptose epimerase
5	GMHEPAT	Cell Envelope Biosynthesis			d8	D-glycero-D-manno-hepose 1-phosphate adenyltransferase
5	GMHEPK	Cell Envelope Biosynthesis			d8	D-glycero-D-manno-heptose 7-phosphate kinase
5	GMHEPPA	Cell Envelope Biosynthesis			d8	D-glycero-D-manno-heptose 1,7-bisphosphate phosphatase
5	S7PI	Cell Envelope Biosynthesis			d8	sedoheptulose 7-phosphate isomerase
6	CHORM	Tyrosine, Tryptophan, and Phenylalanine Metabolism		b		chorismate mutase
7	C141SN	Membrane Lipid Metabolism		b		fatty acid biosynthesis (n-C14:1)

Cluster No.	iJR904	Metabolism	TMI	BN	Duplicate	Enzyme
7	ADMDCr	Arginine and Proline Metabolism			d9	adenosylmethionine decarboxylase
7	MDRPD	Arginine and Proline Metabolism			d9	5-methylthio-5-deoxy-D-ribulose 1-phosphate dehydratase
7	MTAN	Arginine and Proline Metabolism			d9	methylthioadenosine nucleosidase
7	MTRI	Arginine and Proline Metabolism			d9	5-methylthioribose-1-phosphate isomerase
7	MTRK	Arginine and Proline Metabolism			d9	5-methylthioribose kinase
7	SPMS	Arginine and Proline Metabolism			d9	spermidine synthase
7	UNK3	Arginine and Proline Metabolism			d9	2-keto-4-methylthiobutyrate transamination
7	METAT	Methionine Metabolism			d9	methionine adenosyltransferase
8	GALU	Alternate Carbon Metabolism		b		UTP-glucose-1-phosphate uridylyltransferase
9	ASNS2	Alanine and aspartate metabolism		b		asparagine synthetase
10	ASPCT	Purine and Pyrimidine Biosynthesis		b	d10	aspartate carbamoyltransferase
10	OMPDC	Purine and Pyrimidine Biosynthesis			d10	orotidine-5'-phosphate decarboxylase
10	DHORTS	Purine and Pyrimidine Biosynthesis			d10	dihydroorotase
10	ORPT	Purine and Pyrimidine Biosynthesis			d10	orotate phosphoribosyltransferase
11	ANPRT	Tyrosine, Tryptophan, and Phenylalanine Metabolism		b	d11	anthranilate phosphoribosyltransferase
11	ANS	Tyrosine, Tryptophan, and Phenylalanine Metabolism			d11	anthranilate synthase
11	IGPS	Tyrosine, Tryptophan, and Phenylalanine Metabolism			d11	indole-3-glycerol-phosphate synthase
11	PRAIi	Tyrosine, Tryptophan, and Phenylalanine Metabolism			d11	phosphoribosylanthranilate isomerase (irreversible)
11	TRPS3	Tyrosine, Tryptophan, and Phenylalanine Metabolism				tryptophan synthase (indoleglycerol phosphate)
12	PIt2r	Transport, Extracellular		b		phosphate reversible transport via symport
13	CLPNS_EC	Membrane Lipid Metabolism	0.7586	b		cardiolipin synthase (E. coli)
13	EDTXS1	Cell Envelope Biosynthesis	0.7509		d12	endotoxin synthesis (lauroyl transferase)
13	EDTXS2	Cell Envelope Biosynthesis			d12	endotoxin synthesis (myristoyl transferase)
13	LPADSS	Cell Envelope Biosynthesis			d12	lipid A disaccharide synthase
13	LPSSYN_EC	Cell Envelope Biosynthesis			d12	lipopolysaccharide synthesis (Ecoli)
13	MOAT	Cell Envelope Biosynthesis			d12	3-deoxy-D-manno-octulosonic acid transferase
13	MOAT2	Cell Envelope Biosynthesis			d12	3-deoxy-D-manno-octulosonic acid transferase
13	TDSK	Cell Envelope Biosynthesis			d12	tetraacyldisaccharide 4' kinase
13	USHD	Cell Envelope Biosynthesis			d12	UDP-sugar hydrolase
13	NADS1	Cofactor and Prosthetic Group Biosynthesis	0.6648		d13	NAD synthase (NH3)
13	NNAT	Cofactor and Prosthetic Group Biosynthesis			d13	nicotinate-nucleotide adenylyltransferase
13	NNDPR	Cofactor and Prosthetic Group Biosynthesis			d13	nicotinate-nucleotide diphosphorylase (carboxylating)
13	QULNS	Cofactor and Prosthetic Group Biosynthesis			d13	quinolinate synthase
14	G5SD	Arginine and Proline Metabolism		b	d14	glutamate-5-semialdehyde dehydrogenase
14	GLU5K	Arginine and Proline Metabolism			d14	glutamate 5-kinase
15	C160SN	Membrane Lipid Metabolism	1.4045	b		fatty acid biosynthesis (n-C16:0)
15	PGPP_EC	Membrane Lipid Metabolism	1.2649		d15	phosphatidylglycerol phosphate phosphatase (E. coli)
15	PGSA_EC	Membrane Lipid Metabolism			d15	phosphatidylglycerol synthase (E. coli)
15	C140SN	Membrane Lipid Metabolism	1.2299			fatty acid biosynthesis (n-C14:0)
15	ACCOACr	Membrane Lipid Metabolism	1.2186		d16	acetyl-CoA carboxylase, reversible reaction
15	MCOATA	Membrane Lipid Metabolism			d16	malonyl-CoA-ACP transacylase
16	ADSL1r	Purine and Pyrimidine Biosynthesis		b	d17	adenylsuccinate lyase
16	ADSS	Purine and Pyrimidine Biosynthesis			d17	adenylosuccinate synthase
17	IMPD	Purine and Pyrimidine Biosynthesis	2.8402	b		IMP dehydrogenase
17	ADCL	Cofactor and Prosthetic Group Biosynthesis	2.6849		d18	4-aminobenzoate synthase
17	ADCS	Cofactor and Prosthetic Group Biosynthesis			d18	4-amino-4-deoxychorismate synthase
17	DHFS	Cofactor and Prosthetic Group Biosynthesis			d18	dihydrofolate synthase
17	DHNPA2	Cofactor and Prosthetic Group Biosynthesis			d18	dihydroneopterin aldolase
17	DHPS2	Cofactor and Prosthetic Group Biosynthesis			d18	dihydropteroate synthase
17	DNMPPA	Cofactor and Prosthetic Group Biosynthesis			d18	dihydroneopterin monophosphate dephosphorylase
17	DNTPPA	Cofactor and Prosthetic Group Biosynthesis			d18	dihydroneopterin triphosphate pyrophosphatase
17	GTPCI	Cofactor and Prosthetic Group Biosynthesis			d18	GTP cyclohydrolase I
17	HPPK2	Cofactor and Prosthetic Group Biosynthesis			d18	6-hydroxymethyl-dihydropterin pyrophosphokinase
17	GCALDD	Folate Metabolism			d18	glycolaldehyde dehydrogenase
17	GMPS2	Purine and Pyrimidine Biosynthesis	2.6683			GMP synthase

Cluster No.	iJR904	Metabolism	TMI	BN	Duplicate	Enzyme
17	DHFR	Cofactor and Prosthetic Group Biosynthesis	2.5529			dihydrofolate reductase
17	CHORS	Tyrosine, Tryptophan, and Phenylalanine Metabolism	2.5483		d19	chorismate synthase
17	DDPA	Tyrosine, Tryptophan, and Phenylalanine Metabolism			d19	3-deoxy-D-arabino-heptulosonate 7-phosphate synthetase
17	DHQD	Tyrosine, Tryptophan, and Phenylalanine Metabolism			d19	3-dehydroquinate dehydratase
17	DHQS	Tyrosine, Tryptophan, and Phenylalanine Metabolism			d19	3-dehydroquinate synthase
17	PSCVT	Tyrosine, Tryptophan, and Phenylalanine Metabolism			d19	3-phosphoshikimate 1-carboxyvinyltransferase
17	SHK3Dr	Tyrosine, Tryptophan, and Phenylalanine Metabolism			d19	shikimate dehydrogenase
17	SHKK	Tyrosine, Tryptophan, and Phenylalanine Metabolism			d19	shikimate kinase
17	AICART	Purine and Pyrimidine Biosynthesis	2.5085		d20	phosphoribosylaminoimidazolecarboxamide formyltransferase
17	IMPC	Purine and Pyrimidine Biosynthesis			d20	IMP cyclohydrolase
17	ADSL2r	Purine and Pyrimidine Biosynthesis	2.4184		d21	adenylosuccinate lyase
17	AIRC2	Purine and Pyrimidine Biosynthesis			d21	phosphoribosylaminoimidazole carboxylase
17	GLUPRT	Purine and Pyrimidine Biosynthesis			d21	glutamine phosphoribosyldiphosphate amidotransferase
17	PRAGSr	Purine and Pyrimidine Biosynthesis			d21	phosphoribosylglycinamide synthase
17	PRAIS	Purine and Pyrimidine Biosynthesis			d21	phosphoribosylaminoimidazole synthase
17	PRASCS	Purine and Pyrimidine Biosynthesis			d21	phosphoribosylaminoimidazolesuccinocarboxamide synthase
17	PRFGS	Purine and Pyrimidine Biosynthesis			d21	phosphoribosylformylglycinamidine synthase
17	AIRC3	Purine and Pyrimidine Biosynthesis			d21	phosphoribosylaminoimidazole carboxylase (mutase reaction)
18	NADK	Cofactor and Prosthetic Group Biosynthesis		b		NAD kinase
19	GARFT	Purine and Pyrimidine Biosynthesis		b		phosphoribosylglycinamide formyltransferase
20	ACGK	Arginine and Proline Metabolism		b	d22	acetylglutamate kinase
20	ACGS	Arginine and Proline Metabolism			d22	N-acetylglutamate synthase
20	AGPR	Arginine and Proline Metabolism			d22	N-acetyl-g-glutamyl-phosphate reductase
20	ACKr	Pyruvate metabolism			d23	acetate kinase
20	PTAr	Pyruvate metabolism			d23	phosphotransacetylase
21	EDA	Pentose phosphate pathway		b	d24	2-dehydro-3-deoxy-phosphogluconate aldolase
21	EDD	Pentose phosphate pathway			d24	6-phosphogluconate dehydratase
21	PFL	Pyruvate metabolism				pyruvate formate lyase
22	PRPPS	Histidine Metabolism		b		phosphoribosylpyrophosphate synthetase
23	CBMK	Putative		b		carbamate kinase
24	PPC	Anaplerotic reactions		b		phosphoenolpyruvate carboxylase
25	GK1	Nucleotide Salvage Pathways		b		guanylate kinase (GMP:ATP)
26	RPI	Pentose phosphate pathway		b		ribose-5-phosphate isomerase
27	UMPK	Nucleotide Salvage Pathways		b		UMP kinase
28	O2t	Transport, Extracellular	1.9421	b		o2 transport via diffusion
28	ACONT	Citrate Cycle (TCA)	1.9403		d25	aconitase
28	CS	Citrate Cycle (TCA)			d25	citrate synthase
28	CYTBO3	Oxidative phosphorylation	1.8992			cytochrome oxidase bo3 (ubiquinol-8: 2.5 protons)
28	SUCD1i	Citrate Cycle (TCA)	1.6641			succinate dehydrogenase
28	SUCD4	Oxidative phosphorylation	1.6029			succinate dehydrogenase
28	FUM	Citrate Cycle (TCA)	1.5716			fumarase
28	ATPS4r	Oxidative phosphorylation	1.4069			ATP synthase (four protons for one ATP)
28	MDH	Citrate Cycle (TCA)	1.0166			malate dehydrogenase
29	PGMT	Alternate Carbon Metabolism	1.5347	b		phosphoglucomutase
29	N/A (HSST or SHSL1)	Methionine Metabolism	1.4886			O-succinylhomoserine lyase
29	PGAMT	Cell Envelope Biosynthesis	1.4872			phosphoglucosamine mutase
29	URIDK2r	Nucleotide Salvage Pathways	0.8324			uridylate kinase (dUMP)
29	PUNP1	Nucleotide Salvage Pathways	0.7181			purine-nucleoside phosphorylase (adenosine)
30	H2Ot	Transport, Extracellular				H2O transport via diffusion
30	CO2t	Transport, Extracellular		b		CO2 transporter via diffusion
31	PPCK	Anaplerotic reactions		b		phosphoenolpyruvate carboxykinase
32	FBA	Glycolysis/Gluconeogenesis		b	d26	fructose-bisphosphate aldolase
32	PFK	Glycolysis/Gluconeogenesis			d26	phosphofructokinase

Cluster No.	iJR904	Metabolism	TMI	BN	Duplicate	Enzyme
33	HCO3E	Unassigned				HCO3 equilibration reaction
33	GLNS	Glutamate metabolism		b		glutamine synthetase
34	PPM	Alternate Carbon Metabolism		b		phosphopentomutase
35	CYTK1	Nucleotide Salvage Pathways	1.8828	b		cytidylate kinase (CMP)
35	PSSA_EC	Membrane Lipid Metabolism	1.7844			phosphatidylserine synthase (E. coli)
35	PSD_EC	Membrane Lipid Metabolism	1.7410			phosphatidylserine decarboxylase (E. coli)
35	DASYN_EC	Membrane Lipid Metabolism	1.6845			CDP-diacylglycerol synthetase (E. coli)
35	PASYN_EC	Membrane Lipid Metabolism	1.6502			phosphatidic acid synthase (E. coli)
35	PPA	Anaplerotic reactions	0.9409			inorganic diphosphatase
36	ASPTA	Alanine and aspartate metabolism				aspartate transaminase
36	GLUDy	Glutamate metabolism		b		glutamate dehydrogenase (NADP)
37	CTPS2	Purine and Pyrimidine Biosynthesis		b		CTP synthase (glutamine)
38	NADH6	Oxidative phosphorylation		b		NADH dehydrogenase (ubiquinone-8 & 3.5 protons)
39	GHMT2	Glycine and Serine Metabolism	1.0449	b		glycine hydroxymethyltransferase
39	MTHFC	Folate Metabolism	0.9404		d27	methenyltetrahydrofolate cyclohydrolase
39	MTHFD	Folate Metabolism			d27	methylenetetrahydrofolate dehydrogenase (NADP)
39	PGCD	Glycine and Serine Metabolism	0.8755		d28	phosphoglycerate dehydrogenase
39	PSERT	Glycine and Serine Metabolism			d28	phosphoserine transaminase
39	PSP_L	Glycine and Serine Metabolism			d28	phosphoserine phosphatase (L-serine)
39	TRDR	Oxidative phosphorylation	0.6405			thioredoxin reductase (NADPH)
40	G6PDH2r	Pentose phosphate pathway		b	d29	glucose 6-phosphate dehydrogenase
40	PGL	Pentose phosphate pathway			d29	6-phosphogluconolactonase
41	PPND	Tyrosine, Tryptophan, and Phenylalanine Metabolism	1.6341	b	d30	prephenate dehydrogenase
41	TYRTA	Tyrosine, Tryptophan, and Phenylalanine Metabolism			d30	tyrosine transaminase
41	GLCS1	Glycolysis/Gluconeogenesis	1.6335		d31	glycogen synthase (ADPGlc)
41	GLGC	Glycolysis/Gluconeogenesis			d31	glucose-1-phosphate adenylyltransferase
41	ATPPRT	Histidine Metabolism	1.6009		d32	ATP phosphoribosyltransferase
41	HISTD	Histidine Metabolism			d32	histidinol dehydrogenase
41	HISTP	Histidine Metabolism			d32	histidinol-phosphatase
41	HSTPT	Histidine Metabolism			d32	histidinol-phosphate transaminase
41	IG3PS	Histidine Metabolism			d32	Imidazole-glycerol-3-phosphate synthase
41	IGPDH	Histidine Metabolism			d32	imidazoleglycerol-phosphate dehydratase
41	PRAMPC	Histidine Metabolism			d32	phosphoribosyl-AMP cyclohydrolase
41	PRATPP	Histidine Metabolism			d32	phosphoribosyl-ATP pyrophosphatase
41	PRMICIi	Histidine Metabolism			d32	1-(5-phosphoribosyl)-5-[(5-phosphoribosylamino)methylideneamino)imidazole-4-carboxamide isomerase (irreversible)
41	C181SN	Membrane Lipid Metabolism	1.5990			fatty acid biosynthesis (n-C18:1)
42	A5PISO	Alternate Carbon Metabolism	1.0791	b	d33	arabinose-5-phosphate isomerase
42	KDOCT2	Cell Envelope Biosynthesis			d33	3-deoxy-manno-octulosonate cytidylyltransferase
42	KDOPP	Cell Envelope Biosynthesis			d33	3-deoxy-manno-octulosonate-8-phosphatase
42	KDOPS	Cell Envelope Biosynthesis			d33	3-deoxy -D-manno-octulosonic -acid 8-phosphate
42	C120SN	Membrane Lipid Metabolism			d33	fatty acid biosynthesis (n-C12:0)
42	ALAR	Alanine and aspartate metabolism	1.0582			alanine racemase
42	G1PACT	Cell Envelope Biosynthesis	1.0481		d34	glucosamine-1-phosphate N-acetyltransferase
42	GF6PTA	Cell Envelope Biosynthesis			d34	glutamine-fructose-6-phosphate transaminase
42	UAGDP	Cell Envelope Biosynthesis			d34	UDP-N-acetylglucosamine diphosphorylase
43	ABTA	Arginine and Proline Metabolism	0.9005	b		4-aminobutyrate transaminase
43	GLUDC	Glutamate metabolism	0.8909			glutamate decarboxylase
43	ICDHyr	Citrate Cycle (TCA)	0.6497			isocitrate dehydrogenase (NADP)
44	TKT1	Pentose phosphate pathway	3.2379	b		transketolase
44	TALA	Pentose phosphate pathway	3.2164			transaldolase
44	RPE	Pentose phosphate pathway	3.0078			ribulose 5-phosphate 3-epimerase
44	PGI	Glycolysis/Gluconeogenesis	2.7535			glucose-6-phosphate isomerase
44	TKT2	Pentose phosphate pathway	2.6210			transketolase

Cluster No.	iJR904	Metabolism	TMI	BN	Duplicate	Enzyme
44	TPI	Glycolysis/Gluconeogenesis	2.6085			triose-phosphate isomerase
44	PDH	Glycolysis/Gluconeogenesis	2.5048			pyruvate dehydrogenase
44	GAPD	Glycolysis/Gluconeogenesis	2.4581		d35	glyceraldehyde-3-phosphate dehydrogenase
44	PGK	Glycolysis/Gluconeogenesis			d35	phosphoglycerate kinase
44	GND	Pentose phosphate pathway	2.0632			phosphogluconate dehydrogenase
44	ENO	Glycolysis/Gluconeogenesis	2.0424		d36	enolase
44	PGM	Glycolysis/Gluconeogenesis			d36	phosphoglycerate mutase
44	PYK	Glycolysis/Gluconeogenesis	1.9383			pyruvate kinase
44	GLCpts	Transport, Extracellular	1.7127			D-glucose transport via PEP:Pyr PTS
