# Supplementary material for: Framework for network modularization and Bayesian network analysis to investigate the perturbed metabolic network
Source: BMC Syst Biol. 2011 Dec 14;5(Suppl 2):S14. doi: 10.1186/1752-0509-5-S2-S14 (PMC3287480; doi:10.1186/1752-0509-5-S2-S14)

Figure S1. Local scale Bayesian networks of clusters from wild-type and *lpdA* mutant of *E. coli*. Full information on reactions, including their total mutual information (TMI) values, is available in Table S1.

Bayesian network of Cluster 1. Abbreviations are: ADSK, adenylyl-sulfate kinase; BiomassFlux, cell growth rate; CYSTL, cystathionine b-lyase; G5SADs, L-glutamate 5-semialdehyde dehydratase; MTHFR2, 5,10-methylenetetrahydrofolate reductase (NADH).

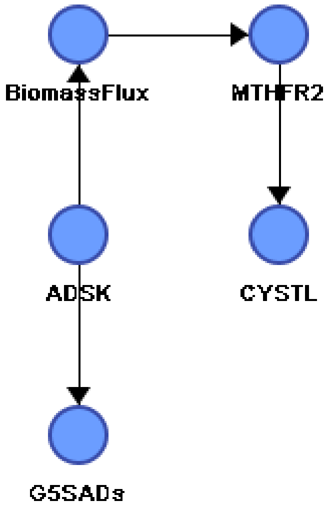

Bayesian network of Cluster 5. Abbreviations are: AGMHE, ADP-D-glycero-D-manno-heptose epimerase; ALAALAr, D-alanine-D-alanine ligase (reversible); DAGK_EC, Diacylglycerol kinase.

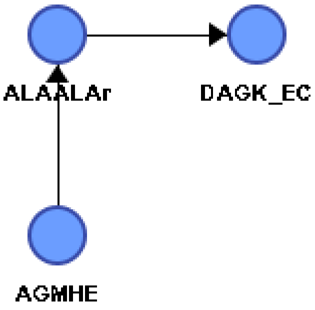

Bayesian network of Cluster 13. Abbreviations are: CLPNS_EC, cardiolipin synthase; EDTXS1, endotoxin synthesis (lauroyl transferase); NADS1, NAD synthase (NH_3).

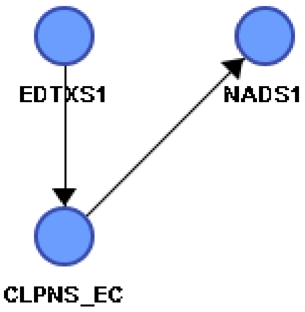

Bayesian network of Cluster 15. Abbreviations are: ACCOACr, acetyl-CoA carboxylase; C140SN, fatty acid biosynthesis (n-C14:0); C160SN, fatty acid biosynthesis (n-C16:0); PGPP_EC, phosphatidylglycerol phosphate phosphatase.

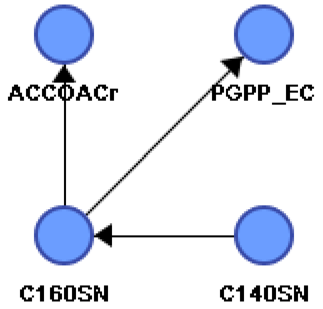

Bayesian network of Cluster 17. Abbreviations are: ADCL, 4-aminobenzoate synthase; ADSL2r, adenylosuccinate lyase; AICART, phosphoribosylaminoimidazolecarboxamide formyltransferase; CHORS, chorismate synthase; DHFR, dihydrofolate reductase; GMPS2, GMP synthase; IMPD, IMP dehydrogenase.

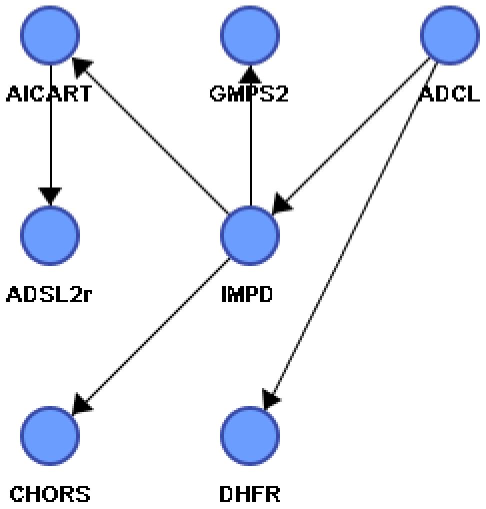

Bayesian network of Cluster 28. Abbreviations are: ACONT, aconitase; ATPS4r, ATP synthase; CYTBO3, cytochrome oxidase bo3; FUM_Rxn, fumarase; MDH, malate dehydrogenase; O2t, O_2 transport via diffusion; SUCD1i, succinate dehydrogenase; SUCD4, succinate dehydrogenase.

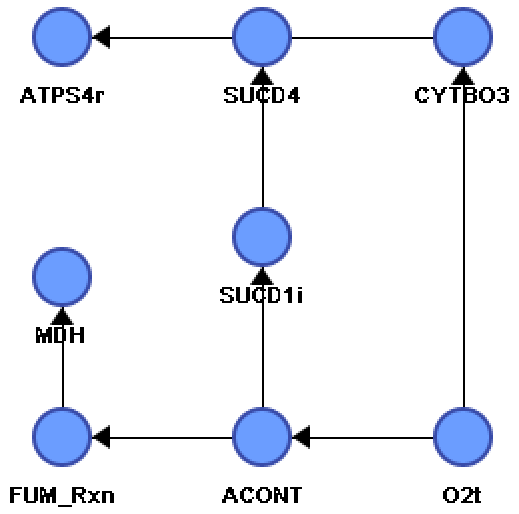

Bayesian network of Cluster 29. Abbreviations are: HSST or SHSL1, homoserine O-succinyltransferase or O-succinylhomoserine lyase (L-cysteine); PGAMT, phosphoglucosamine mutase; PGMT, phosphoglucomutase; PUNP1, purine-nucleoside phosphorylase (adenosine); URIDK2r, uridylate kinase (dUMP).

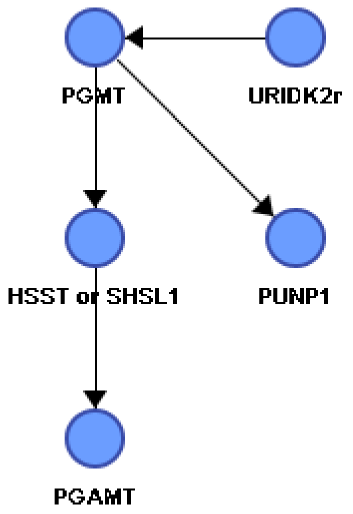

Bayesian network of Cluster 35. Abbreviations are: CYTK1, cytidylate kinase (CMP); DASYN_EC, CDP-diacylglycerol synthetase; PASYN_EC, phosphatidic acid synthase; PPA_Rxn, inorganic diphosphatase; PSD_EC, phosphatidylserine decarboxylase; PSSA_EC, phosphatidylserine synthase.

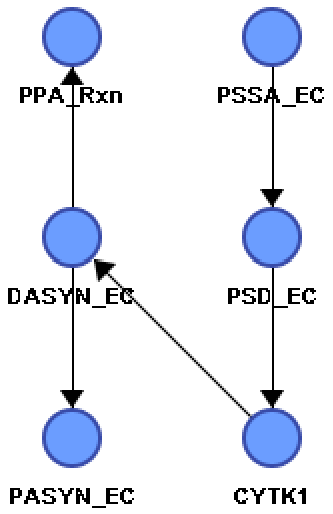

Bayesian network of Cluster 39. Abbreviations are: GHMT2, glycine hydroxymethyltransferase; MTHFC, methenyltetrahydrofolate cyclohydrolase; PGCD, phosphoglycerate dehydrogenase; TRDR, thioredoxin reductase (NADPH).

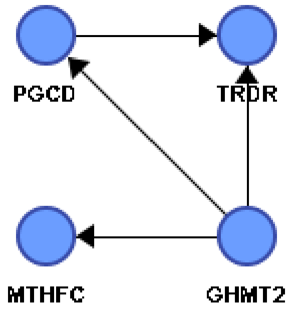

Bayesian network of Cluster 41. Abbreviations are: ATPPRT, ATP phosphoribosyltransferase; C181SN, fatty acid biosynthesis (n-C18:1); GLCS1, glycogen synthase; PPND, prephenate dehydrogenase.

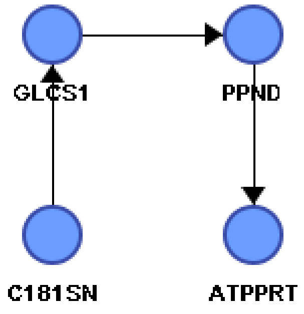

Bayesian network of Cluster 42. Abbreviations are: A5PISO, arabinose-5-phosphate isomerase; ALAR, alanine racemase; G1PACT, glucosamine-1-phosphate N-acetyltransferase.

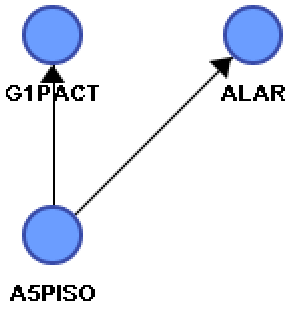

Bayesian network of Cluster 43. Abbreviations are: ABTA, 4-aminobutyrate transaminase; GLUDC, glutamate decarboxylase; ICDHyr, isocitrate dehydrogenase (NADP).

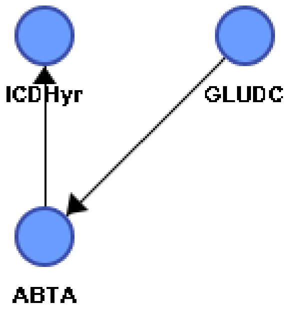

Bayesian network of Cluster 44. Abbreviations are: ENO, enolase; GAPD, glyceraldehyde-3-phosphate dehydrogenase; GLCpts, D-glucose transport via PEP:Pyr PTS; GND, phosphogluconate dehydrogenase; PDH, pyruvate dehydrogenase; PGI, glucose-6-phosphate isomerase; PYK, pyruvate kinase; RPE, ribulose 5-phosphate 3-epimerase; TALA, transaldolase; TKT1, transketolase; TKT2, transketolase; TPI, triose-phosphate isomerase.

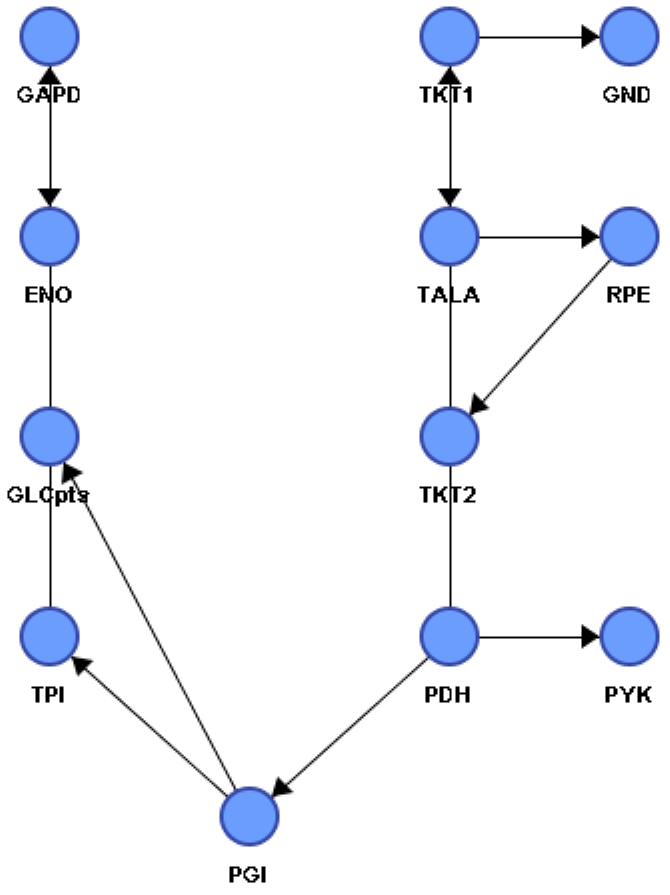

Supplement: Additional file 2 — Figure S1. Local scale Bayesian networks of clusters from wild-type and lpdA mutant of E. coli. [file 1752-0509-5-S2-S14-S2.pdf]
